# High Blood Pressure among Students in Public and Private Schools in Maceió, Brazil

**DOI:** 10.1371/journal.pone.0142982

**Published:** 2015-11-23

**Authors:** Haroldo S. Ferreira, Glícia Maris A. Lúcio, Monica L. Assunção, Bárbara Coelho V. Silva, Juliana S. Oliveira, Telma Maria M. T. Florêncio, Amandio Aristides R. Geraldes, Bernardo L. Horta

**Affiliations:** 1 Faculty of Nutrition, Federal University of Alagoas, Maceió, Alagoas, Brazil; 2 Postgraduate Program in Nutrition, Faculty of Nutrition, Federal University of Alagoas, Maceió, Alagoas, Brazil; 3 Academic Center of Vitória, Federal University of Pernambuco, Vitória de Santo Antão, Pernambuco, Brazil; 4 Education Center, Federal University of Alagoas, Maceió, Alagoas, Brazil; 5 Postgraduate Program in Epidemiology, Federal University of Pelotas, Pelotas, Rio Grande do Sul, Brazil; National Cardiovascular Center Hospital, JAPAN

## Abstract

The prevalence of hypertension in childhood is increasing, and investigation of its distribution is important for planning timely interventions. This study assessed the prevalence of high blood pressure (HBP) and associated factors in students between 9 and 11 years of age enrolled in public and private schools in Maceió, Brazil. A cross-sectional study was performed in a probabilistic sample of students (10.3 ± 0.5 years). The students were selected from a systematic sampling of 80 schools (40 public and 40 private). To maintain similar proportions of students existing in public and private schools in Maceió, 21 and 14 students were randomly selected from each public and private school, respectively. The prevalence ratio (PR) was estimated using Poisson regression. A total of 1,338 students were evaluated (800 from public schools and 538 from private schools). No differences were observed between school types in terms of student age and gender (*p* > 0.05). The prevalence of obesity (19.9% vs. 9.0%; PR = 2.2; 95% CI = 1.67–2.92) and hypertension (21.2% vs. 11.4%; PR = 1.86; 95% CI = 1.45–2.40) were higher in private schools. The association between high blood pressure and type of school (public or private) remained statistically significant even after adjustment for obesity (PR = 1.53; 95% CI = 1.19–1.97). In conclusion: (a) students from private schools have higher socioeconomic status, BMI, and HBP prevalence compared to those of public school; (b) among the evaluated students, the prevalence of obesity only partially explained the higher prevalence of high blood pressure among students from private schools. Other factors related to lifestyle of children from private schools may explain the higher prevalence of HBP. This results show the need to implement measures to promote healthy lifestyles in the school environment, since children with HBP are more likely to become hypertensive adults. Therefore, early detection and intervention in children with HBP is an important action for the prevention of hypertension in adulthood.

## Introduction

The increasing prevalence of obesity worldwide is concerning owing to its association with multiple comorbid conditions, especially cardiovascular diseases such as hypertension [1]. Hypertension is a key public health issue, accounting for 7.6 million deaths worldwide annually, 80% of which occur in developing countries such as Brazil. About 54% and 47% of stroke and myocardial infarction cases, respectively, worldwide are associated with hypertension [2].

Studies on the prevalence of high blood pressure (HBP) in children are important for revealing the magnitude of the issue and identifying target groups for timely intervention. Most studies in Brazil have been performed in school settings. The preference for school surveys may be explained by the logistic ease of sample selection. Magalhães et al. [3] observed that 19 of 21 articles on HBP prevalence in Brazilian children and adolescents published between 1995 and 2010, were performed on schoolchildren. However, about half of these studies evaluated only students from a single type of school (public or private). The others used samples from both types of schools, but they did not stratify the analysis by type of school.

There are marked socioeconomic disparities among students from public and private schools. There appear to be differences in the prevalence of obesity (and consequently hypertension) according to the socioeconomic characteristics of the population [4–6]. Using data from a longitudinal study, Miyazaki and Stack [7], reported an inverse relationship between body mass index (BMI) and socioeconomic status, except for black male children. Bammann et al. [8] investigated the association between socioeconomic status (SES) and childhood overweight in Europe. In five of the eight studied regions (Belgium, Estonia, Germany, Spain, and Sweden), the prevalence of childhood overweight followed an inverse SES gradient. In the other three regions (Cyprus, Hungary, and Italy), no association between SES and childhood overweight was found.

In light of these conflicting reports, evaluating only one school type (public or private) would not yield results representative of the prevailing situation in the entire population. Furthermore, aggregated data analysis could result in the underestimation or overestimation of the prevalence of overweight/hypertension depending on the direction of the association between socioeconomic status and overweight/hypertension.

To our knowledge, no previous studies have investigated the association between socioeconomic level and hypertension in schoolchildren in Maceió, the capital of Alagoas, one of the poorest states in Brazil and that has been undergoing a rapid process of nutritional transition [9].

This study assessed the prevalence of high blood pressure and associated factors (socioeconomic and anthropometric) in students between 9 and 11 years of age enrolled in public and private schools in Maceió, Brazil.

## Materials and Methods

### Study population and sampling

A cross-sectional study was carried out between October 2012 and May 2013. The sample consisted of male and female children aged between 9 and 11 years enrolled in elementary schools in Maceió, state of Alagoas.

According to the Brazilian Educational Census [10], the study population was estimated in 31,488 students. Of these, about two of three children were enrolled in public schools and one of every three in private schools. The sample size was calculated using Epi Info StatCalc software version 7.1.3.10 using the following parameters: 9.4% HBP prevalence [11], sampling error of 2.5 percentage points, design effect equal to 2.0, and number of clusters equal to 80 schools. A 95% confidence interval (CI) required 1,040 individuals; increasing this minimum by 30% for sampling losses meant that a total of 1,352 students were required for this study.

A cluster randomization sampling was used, in which each school was regarded as a cluster. To ensure geographic homogeneity across different localities in the municipality, a systematic sample of 80 schools (40 public and 40 private) was obtained from a listing (http://www.inep.gov.br/) of the educational institutions existing throughout the city. To maintain the same proportion of students enrolled in each type of school within the sample (2/3 from public and 1/3 from private schools), 21 students and 14 students from each public and private school were randomly selected, resulting in an estimated sample of 1,400 students (840 public and 560 private school students). These students were randomly selected from a list provided by the staff of the respective establishment.

### Data collection

Data were collected by a trained team. To assess blood pressure, portable digital monitors Omron HEM 705 CP (Omron, Tokyo, Japan) were used, which were validated for use on individuals within the age group being studied [12] and whose accuracy was verified weekly by comparing the results to those obtained by an auscultation method using a mercury column sphygmomanometer (Wan Ross, USA) and a clinical stethoscope (BD, Brazil). Two cuff sizes were used, depending on the size of the child's circumference arm: one for an arm circumference of 10–19 cm and another for an arm circumference of 18–26 cm.

Blood pressure was measured twice, in the morning and, no less than 30 minutes after breakfast, when children had empty bladders, after at least 5 minutes of rest, sitting, with back support and feet on the ground, without moving or talking [13]. The first measurement was performed 5 min after commencing the interview and the second was performed after a minimum interval of 10 min. Whenever there was a discrepancy greater than 5 mmHg between the two values, a third measurement was performed and a mean value calculated after disregarding the value showing the largest deviation.

For the purposes of the analysis, the mean value of the two assessments was compared to reference data [14], expressed in percentiles, considering gender, age, and stature-for-age percentile of the child.

High blood pressure was defined as systolic and/or diastolic blood pressure higher than or equal to percentile 95, according to the rating proposed by the fourth report on the diagnosis, evaluation, and treatment of high blood pressure in children and adolescents [14].

To obtain information on socioeconomic and demographic variables, specific questionnaires were sent with students for their parents to complete. Completed surveys were returned to the researchers, together with informed consent forms. The variables included:

1. Home ownership: “yes” or “no.”
2. Education level of household head: number of completed years of schooling.
3. Number of household members.
4. Per capita income: income of the family members (including salary, government programs, retirements, and donations) divided by the total number of household members. The value obtained was converted into US dollars. At the time of the study, the dollar/real ratio was 1:2.14.

Information on skin color was collected by the interviewer through direct observation.

Anthropometric variables were obtained according to Frisancho’s recommendations [15] and analyzed according to the World Health Organization reference data [16]. Weight was measured with Tanita® Digital Scale HD313 (Itin Scale, Brooklyn, USA) scales. Height was measured using stadiometers Seca® (Seca, Hamburg, Germany). Measurements were taken to the nearest 0.1kg, and 0.1 cm, respectively. Z scores of BMI per age and gender (BMI-for-age; kg/m^2^) were obtained using the AnthroPlus application [17]. Obesity was defined as BMI z-score at least two standard deviations (SD) above the mean.

### Data analysis

Data were entered twice in a form designed on Google Docs®; after comparing both files, errors were identified and corrected. Data analysis was performed using Stata® 12.0 (Stata Corp., College Station, TX, USA).

Absolute and relative frequencies were used to characterize the sample. Chi-squared tests were used to compare proportions (Fisher’s exact test for frequencies equal to or fewer than five cases), and Student’s t-tests were used to compare means. Prevalence ratio (PR) and corresponding 95% confidence intervals (95% CIs) were used as a measure of association. PR was estimated using Poisson regression with robust adjustment of variance. This procedure was also used to adjust the prevalence of HBP according to the prevalence of obesity.

The research protocol was approved by the Ethics Committee of the Federal University of Alagoas (Case no. 017299/2011-43). Only children who returned informed consent forms in writing duly signed by their respective guardians were included in the study.

## Results

This study included 1,338 students, 800 and 538 from public and private schools, respectively. Anthropometric data, blood pressure, skin color, gender, and age were obtained from all participants. However, due to unreturned parent/representative questionnaires, information on socioeconomic and demographic indicators was unavailable for 34.2% of students from public schools and 7.1% of students from private schools.

In spite of the high proportion of students from public schools who did not return the questionnaire, we believe that these losses did not introduce a selection bias. In the public schools, where the percentage of loss was higher, the mean z scores of BMI-for-age (0.14 vs. 0.17; p = 0.76), height-for-age index (-0.04 vs -0.15; p = 0.10), mean systolic blood pressure (104.4 vs. 103.4 mmHg, p = 0.20) and diastolic blood pressure (64.4 vs. 63.7 mmHg; p = 0.28), were similar among those subjects who returned and did not return the questionnaire.

Table 1 shows that the proportion of male subjects was similar in public and private schools. In public schools, 80.7% of students classified by the interviewer were black or brown, while in private schools this proportion was 54.1% (P < 0.001). Among students enrolled in public schools, there was also a higher proportion of families who did not own a home (47.0% vs. 28.2%), with heads of household with less than eight years of schooling (76.3% vs. 31.3%), with more than four household members (49.7% vs. 27.2%), and with a per capita family income of less than $2 per day (37.9% vs. 3.7%). On the other hand, mean age of the study participants was independent of type of school.

**Table 1:** Socioeconomic, demographic, nutritional, and health characteristics of elementary school children according to type of school. Maceió (Alagoas), Brazil, 2013.

Variable	Category	Total n (%)	Type of School		*p*-value (χ^2^)
			Public	Private	
Gender	Male	639 (47.8)	378 (47.3)	261 (48.5)	0.650
	Female	699 (52.2)	422 (52.7)	277 (51.5)	
Skin color	White	386 (30.1)	147 (19.3)	239 (45.9)	<0.001
	Black/brown	895 (69.9)	613 (80.7)	282 (54.1)	
Home ownership	Yes	564 (62.3)	241 (53.0)	323 (71.8)	<0.001
	No	341 (37.7)	214 (47.0)	127 (28.2)	
Schooling of household head (years)	≤8	339 (52.5)	232 (76.3)	107 (31.3)	<0.001
	>8	307 (47.5)	72 (23.7)	235 (68.7)	
Number of household members	≤4	576 (61.9)	226 (50.3)	350 (72.8)	<0.001
	>4	354 (38.1)	223 (49.7)	131 (27.2)	
Per capita income (<US $2/day)	Yes	154 (20.5)	140 (37.9)	14 (3.7)	<0.001
	No	598 (79.5)	229 (62.1)	369 (96.3)	
Obesity (BMI-for-age >2 SD)	Yes	179 (13.4)	72 (9.0)	107 (19.9)	<0.001
	No	1157 (86.6)	727 (91.0)	430 (80.1)	
High blood pressure	Yes	205 (15.3)	91 (11.4)	114 (21.2)	<0.001
	No	1133 (84.7)	709 (88.6)	424 (78.8)	

χ^2^: chi-square test; BMI-for-age >2 SD: Body Mass Index-for-age > 2 standard deviation

The prevalence of obesity (19.9% vs. 9.0%) and HBP (21.2% vs. 11.4%) was higher among students enrolled in private schools. Table 2 shows that the prevalence of HBP was associated only with family per capita income below $2/day and obesity.

**Table 2:** Prevalence, prevalence ratio (PR), crude and adjusted confidence intervals (95% CI) of the association between high blood pressure and independent variables among schoolchildren from public and private schools in Maceió (Alagoas), Brazil, 2013.

Variable	Category	Public school		Private school		Total		
		%	PR (95% CI)	%	PR (95% CI)	%	PR (95% CI)	Adjusted PR^a^ (95% CI)
Gender	Male	10.8	0.92 (0.6–1.3)	21.5	1.02 (0.7–1.4)	15.2	0.98 (0.8–1.3)	0.83 (0.65–1.06)
	Female	11.8	1	20.9	1	15.5	1	1
Skin color	White	11.6	1.07 (0.7–1.8)	19.7	0.88 (0.6–1.2)	16.6	1.15 (0.9–1.5)	1.01 (0.77–1.32)
	Black/brown	10.8	1	22.3	1	14.4	1	1
Home ownership	Yes	10.8	0.80 (0.5–1.3)	23.5	1.57 (1.0–2.5)	18.1	1.28 (0.9–1.8)	1.18 (0.88–1.60)
	No	13.5	1	15.0	1	14.1	1	1
Schooling of household head (years)	≤8	12.9	1	13.1	1	13.0	1	1
	>8	15.3	1.18 (0.62–2.24)	20.8	1.59 (0.9–2.8)	19.5	1.51 (1.0–2.2)	1.30 (0.91–1.84)
Number of household members	≤4	11.9	1.02 (0.6–1.7)	19.7	0.83 (0.6–1.2)	16.7	1.03 (0.8–1.4)	0.94 (0.71–1.25)
	>4	11.7	1	23.7	1	16.1	1	1
Per capita income (< US $2/day)	Yes	12.1	1	0.0	nc	11.0	1	1
	No	11.8	0.97 (0.55–1.72)	21.9*	1	18.1	1.64 (1.1–2.6)	1.42 (0.88–2.29)
Obesity (BMI-for-age >2 SD)	Yes	36.1	4.10 (2.8–6.0)	48.6	3.37 (2.5–4.6)	43.6	4.00 (3.2–5.1)	b
	No	8.8	1	14.4		10.9	1	-

PR = prevalence ratio; 95% CI = confidence interval 95%. nc = no calculated due to absence of cases in one of the conditions.

*p < 0.05 (Fisher's exact test)

a. Adjusted for obesity

b. Adjustment variable.

BMI-for-age >2 SD: Body Mass Index-for-age > 2 standard deviation

The association of obesity with high blood pressure was independent of the type of school. Among students from the public school, the prevalence of HBP was 4.10 (95% CI = 2.79–6.04) times higher among obese subjects, whereas in private school the prevalence ratio was 3.37 (95% CI = 2.49–4.56).

The association between high blood pressure and type of school (public or private) remained statistically significant even after adjustment for obesity (PR = 1.53; 95% CI = 1.19–1.97).

## Discussion

The HBP prevalence observed in this study (15.3%) is within the range of values reported in other studies performed in different regions of the country over the last decade, with prevalence ranging from 2.3% to 44.7% [18–25]. This broad range is likely due to differences in epidemiological characteristics of the respective samples and by differences in methodological procedures. According to Pinto et al. [25], factors such as sample age, number of visits, number of blood pressure assessments per visit, and interval between assessments greatly contribute to variation in the prevalence. They highlight the fact that differences in methodologies used to diagnose hypertension make it difficult to compare results across studies.

Subsequent blood pressure measurements after the second visit tended to be lower due to decreased patient anxiety [14]. As a consequence, studies measuring blood pressure over 3 different days [22] or, alternatively, using values from the 3^rd^ assessment during a single visit [19], observed lower values. In one of these studies [22], performed in students between 7 and 14 years of age (Barbacena, MG, Southeastern Brazil), the prevalence of hypertension decreased from 16.6% in the first visit to 2.5% in the third visit.

The prevalence of HBP in this study was similar to reports from surveys of other northeastern Brazilian cities, and which also measured blood pressure on a single visit [26, 27]. In a study performed in João Pessoa (PB) [26] with a random sample of 750 students, the prevalence of HBP was 13.6%. Another study performed in Recife (PE) [27] observed that 17.3% of the studied population had high blood pressure.

A previous survey performed in Maceió [11], carried out 12 years ago, observed a prevalence of 9.4%, suggesting that the prevalence of high blood pressure is increasing. As described by Ferreira et al. [9], the State of Alagoas has been going through a nutritional transition process, in which the prevalence of obesity in children, a major risk factor for hypertension [28], has been growing rapidly. In the previous survey performed in Maceió [11], obesity was defined by a BMI-for-age higher than or equal to the 85^th^ percentile. In our study, the diagnosis was established by a BMI-for-age higher than 2 SD. Using a lower cut-off point (BMI-for-age >1 SD) to allow for a comparison with the values obtained from the 85^th^ percentile, we observed that prevalence of excess weight went from 13.7% in 2001 [11] to 31.0% in 2013 (this study).

Considering the administrative dependence of the educational establishment, marked differences were observed in the prevalence of obesity. The frequency of obesity among students attending public schools (9.0%), while concerning, was less than half that of students attending private schools (19.9%). A previous study of 1,616 children and adolescents from a city in Northeastern Brazil (Recife-PE) also found a higher prevalence of obesity among students at higher socioeconomic levels [29]. According to Oliveira et al. [30], this is due to different lifestyles, as regional, socioeconomic, and cultural factors may interfere with individual physical activity patterns. A study of students in public and private schools in São Luís (MA) [30] observed that students at lower socioeconomic level spent less time with television, videos, videogames, and computers than those belonging to wealthy families. Fernandes et al. [31] also observed that the prevalence of overweight was also positively associated with socioeconomic status.

In this investigation, obesity increased the prevalence of high blood pressure by about four times. The association between HBP and obesity has been reported in other studies [11, 23–25, 32, 33]. However, even after controlling for obesity, prevalence of high blood pressure was still higher among students from private schools. Similar results were obtained by Costanzi and associates [18] in a southern Brazilian city.

Other factors such as inadequate food consumption (processed food, food high in sodium) [34, 35] and sedentary lifestyle [36] may explain the prevalence of HBP not attributable to obesity. Other studies designed specifically to explore this issue are necessary to clarify this matter. However, considering the high prevalence of obesity and HBP in the studied population, especially in private schools, it is highly recommended that schools adopt programs to promote healthy lifestyle choices among students, especially those related to eating habits and physical activity patterns.

Socioeconomic differences were observed between students from public and private schools. Of the 4 indicators of low socioeconomic condition (families without home ownership; head of a household with less than 8 years of schooling; more than 4 household members; per capita income below $2/day), the magnitude of the difference in the proportion of families under the poverty threshold (below $2/day) was the most expressive: 3.7% vs. 37.9%. In other words, the proportion of poor people in public schools was 10-fold that in private schools.

In the crude analysis, there was a lower prevalence of HBP among students whose families had per capita income <US $ 2 / day. However, when was made the adjustment for obesity, this relationship lost significance. Nonetheless, obesity, a major risk factor for BPH, differed significantly between students from public and private schools, as well as also significantly differed with regard to family income.

Specific epidemiological studies are important because this relationship differs according to the different contexts where they are performed. Mocanu [37], in a study carried out with 3444 school children of 6–10 years attending 30 schools in northeast Romania, found that high socioeconomic status were associated with increased risk of overweight, corroborating our findings. However, Thibault et al.[38] analysed a sample of 7667 children aged 5–11 years from Aquitaine region (south-west France), and concluded that low socioeconomic level was associated with a higher risk of being overweight or obese, adding that the social determinants should be considered when implementing preventive measures regarding obesity. Obviously, the same is recommended in relation to BPH.

In view of this, the prevalence of HBP may be over- or underestimated if analyses do not separately consider students in private and public schools, respectively. In light of this, we recommended that studies on HBP stratify analysis of students from both networks.

This study has some limitations. a) Blood pressure was measured with oscillometric devices, despite the auscultation remains the recommended method for blood pressure measurement in children under most circumstances. However, many studies have validated the use of such equipment for measuring blood pressure [12, 39, 40]; b) blood pressure was measured in a single occasion, and the diagnosis of hypertension should be based on readings taken on several visits. Most other epidemiological studies on blood pressure in childhood also relied on readings taken on a single occasion [26, 27, 41, 42]. Is worth noting that, under such circumstances, the analysed outcome should be referred as high blood pressure and not as hypertension; c) Our data were cross-sectional and the causal relationship between obesity, for example, and blood pressure cannot be strongly established. However, as argued by Chiolero et al., the benefits of weight loss for blood pressure reduction in children have been shown in both cohort studies and clinical trials [41].

## Conclusions

Students from private schools have higher socioeconomic status, BMI, and HBP prevalence compared to those of public school. Among the evaluated students, the prevalence of obesity only partially explains the prevalence of high blood pressure. Other factors related to specific lifestyle of children from private schools may explain the higher prevalence of HBP not attributable to obesity per si in these children.

In a public health point of view, the data here presented show the need to implement measures to promote healthy lifestyles in the school environment, especially in private establishments. According to Zhang et al. [43], children with HBP are more likely to become hypertensive adults. Therefore, early detection and intervention in children with HBP is an important action for the prevention of hypertension in adulthood.

## Supporting Information

S1 Table The database that was analyzed for preparation of this manuscript was provided in this file. (XLS)

## References

[1] Farajian P, Renti E, Manios Y. Obesity indices in relation to cardiovascular disease risk factors among young adult female students. The British journal of nutrition. 2008;99(4):918–24. 10.1017/S000711450783741X . 17916274

[2] Lawes CM, Vander Hoorn S, Rodgers A. Global burden of blood-pressure-related disease, 2001. Lancet. 2008;371(9623):1513–8. Epub 2008/05/06. 10.1016/s0140-6736(08)60655-8 . 18456100

[3] Magalhaes MG, Oliveira LM, Christofaro DG, Ritti-Dias RM. Prevalence of high blood pressure in Brazilian adolescents and quality of the employed methodological procedures: systematic review. Revista brasileira de epidemiologia = Brazilian journal of epidemiology. 2013;16(4):849–59. . 24896590 10.1590/s1415-790x2013000400005

[4] Fernandes RA, Casonatto J, Christofaro DG, Ronque ER, Oliveira AR, Freitas Junior IF. Riscos para o excesso de peso entre adolescentes de diferentes classes socioeconômicas. Revista da Associacao Medica Brasileira. 2008;54(4):334–8. 18719792 10.1590/s0104-42302008000400019

[5] Wang Y, Monteiro C, Popkin BM. Trends of obesity and underweight in older children and adolescents in the United States, Brazil, China, and Russia. The American journal of clinical nutrition. 2002;75(6):971–7. . 12036801 10.1093/ajcn/75.6.971

[6] Jones-Smith JC, Gordon-Larsen P, Siddiqi A, Popkin BM. Is the burden of overweight shifting to the poor across the globe? Time trends among women in 39 low- and middle-income countries (1991–2008). International journal of obesity. 2012;36(8):1114–20. 10.1038/ijo.2011.179 21912397 PMC3516372

[7] Miyazaki Y, Stack M. Examining individual and school characteristics associated with child obesity using a multilevel growth model. Soc Sci Med. 2015;128:57–66. Epub 2015/01/16. 10.1016/j.socscimed.2014.12.032 . 25589033

[8] Bammann K, Gwozdz W, Lanfer A, Barba G, De Henauw S, Eiben G, et al Socioeconomic factors and childhood overweight in Europe: results from the multi-centre IDEFICS study. Pediatr Obes. 2013;8(1):1–12. Epub 2012/08/14. 10.1111/j.2047-6310.2012.00075.x . 22888012

[9] Ferreira HS, Cesar JA, Assunção ML, Horta BL. Time trends (1992–2005) in undernutrition and obesity among children under five years of age in Alagoas State, Brazil. Cadernos de Saúde Pública. 2013;29:793–800. 23568308

[10] Brasil, Instituto Nacional de Estudos e Pesquisas Educacionais. Censo Educacional 2012. Rio de Janeiro: Ministério da Educação; 2013.

[11] Moura AA, Silva MA, Ferraz MR, Rivera IR. Prevalence of high blood pressure in children and adolescents from the city of Maceio, Brazil. Jornal de pediatria. 2004;80(1):35–40. . 14978547 10.2223/1131

[12] Furusawa EA, Filho UD, Junior DM, Koch VH. Home and ambulatory blood pressure to identify white coat and masked hypertension in the pediatric patient. American journal of hypertension. 2011;24(8):893–7. 10.1038/ajh.2011.72 . 21525965

[13] VI Diretrizes Brasileiras de Hipertensão. Arquivos brasileiros de cardiologia. 2010;95:I–III.

[14] National High Blood Pressure Education Program Working Group on High Blood Pressure in Children Adolescents. The fourth report on the diagnosis, evaluation, and treatment of high blood pressure in children and adolescents. Pediatrics. 2004;114(2 Suppl 4th Report):555–76. . 15286277

[15] Frisancho AR. Anthropometric standards for the assessment of growth and nutritional status Ann Arbor: University of Michigan Press; 1990.

[16] World Health Organization. Growth reference data for 5–19 years Geneva: WHO; 2007.

[17] World Health Organization. AnthroPlus for Personal Computers Manual: Software for Assessing Growth of the World's Children and Adolescents. Geneva: World Health Organization; 2009.

[18] Costanzi CB, Halpern R, Rech RR, Bergmann ML, Alli LR, Mattos AP. Associated factors in high blood pressure among schoolchildren in a middle size city, southern Brazil. Jornal de pediatria. 2009;85(4):335–40. 10.2223/JPED.1913 . 19668903

[19] Borges LM, Peres MA, Horta BL. Prevalência de níveis pressóricos elevados em escolares de Cuiabá, Mato Grosso. Revista de saude publica. 2007;41:530–8. 17589750 10.1590/s0034-89102006005000040

[20] Mazaro IA, Zanolli ML, Antonio MÂ, Morcillo AM, Zambon MP. Obesidade e fatores de risco cardiovascular em estudantes de Sorocaba, SP. Revista da Associacao Medica Brasileira. 2011;57:674–80. 22249548 10.1590/s0104-42302011000600015

[21] Monego ET, Jardim PCBV. Determinantes de risco para doenças cardiovasculares em escolares. Arquivos brasileiros de cardiologia. 2006;87:37–45. 16906268 10.1590/s0066-782x2006001400006

[22] Rezende DF, Scarpelli RAB, Souza GF, Costa JO, Scarpelli AMB, Scarpelli PA, et al Prevalence of systemic hypertension in students aged 7 to 14 years in the municipality of Barbacena, in the State of Minas Gerais, in 1999. Arquivos brasileiros de cardiologia. 2003;81:381–6. 14666280 10.1590/s0066-782x2003001200005

[23] Oliveira AMA, Oliveira AC, Almeida MS, Almeida FS, Ferreira JBC, Silva CEP, et al Fatores ambientais e antropométricos associados à hipertensão arterial infantil. Arquivos Brasileiros de Endocrinologia & Metabologia. 2004;48:849–54. 15761559 10.1590/s0004-27302004000600011

[24] Nogueira PC, Costa RF, Cunha JSN, Silvestrini L, Fisberg M. Pressão arterial elevada em escolares de Santos: relação com a obesidade. Revista da Associacao Medica Brasileira. 2007;53:426–32. 17952352 10.1590/s0104-42302007000500019

[25] Pinto SL, Silva RCR, Priore SE, Assis AM, Pinto EJ. Prevalência de pré-hipertensão e de hipertensão arterial e avaliação de fatores associados em crianças e adolescentes de escolas públicas de Salvador, Bahia, Brasil. Cadernos de Saúde Pública. 2011;27:1065–75. 21710004 10.1590/s0102-311x2011000600004

[26] Queiroz VM, Moreira PV, Vasconcelos TH, Vianna RP. Prevalência e preditores antropométricos de pressão arterial elevada em escolares de João Pessoa—PB. Arquivos brasileiros de cardiologia. 2010;95:629–34. 21225114 10.1590/s0066-782x2010001500011

[27] Gomes BMR, Alves JGB. Prevalência de hipertensão arterial e fatores associados em estudantes de Ensino Médio de escolas públicas da Região Metropolitana do Recife, Pernambuco, Brasil, 2006. Cadernos de Saúde Pública. 2009;25:375–81. 19219245 10.1590/s0102-311x2009000200016

[28] Huang RC, Burrows S, Mori TA, Oddy WH, Beilin LJ. Lifecourse Adiposity and Blood Pressure Between Birth and 17 Years Old. American journal of hypertension. 2015 Epub 2015/01/21. 10.1093/ajh/hpu266 . 25600223

[29] Silva GA, Balaban G, Motta ME. Prevalência de sobrepeso e obesidade em crianças e adolescentes de diferentes condições socioeconômicas. Revista Brasileira de Saúde Materno Infantil. 2005;5:53–9.

[30] Oliveira TC, Silva AAM, Santos CJN, Silva JS, Conceição SIO. Atividade física e sedentarismo em escolares da rede pública e privada de ensino em São Luís. Revista de saude publica. 2010;44:996–1004.21107499

[31] Fernandes RA, Christofaro DG, Cardoso JR, Ronque ER, Freitas Junior IF, Kawaguti SS, et al Socioeconomic status as determinant of risk factors for overweight in adolescents. Ciencia & saude coletiva. 2011;16(10):4051–7. . 22031134 10.1590/s1413-81232011001100010

[32] Souza MG, Rivera IR, Silva MA, Carvalho AC. Relação da obesidade com a pressão arterial elevada em crianças e adolescentes. Arquivos brasileiros de cardiologia. 2010;94:714–9. 20428712 10.1590/s0066-782x2010005000039

[33] Rosaneli CF, Baena CP, Auler F, Nakashima ATA, Netto-Oliveira ER, Oliveira AB, et al Elevated Blood Pressure and Obesity in Childhood: A Cross-Sectional Evaluation of 4,609 Schoolchildren. Arquivos brasileiros de cardiologia. 2014;103:238–44. 25076178 10.5935/abc.20140104 PMC4193071

[34] Seidell JC, Halberstadt J. [Salt and hypertension: a need for reduction on a population level?]. Nederlands tijdschrift voor geneeskunde. 2015;158(0):A8503 Epub 2015/01/08. . 25563778

[35] Hendriksen MA, Hoogenveen RT, Hoekstra J, Geleijnse JM, Boshuizen HC, van Raaij JM. Potential effect of salt reduction in processed foods on health. The American journal of clinical nutrition. 2014;99(3):446–53. Epub 2013/12/18. 10.3945/ajcn.113.062018 . 24335058

[36] Beunza JJ, Martinez-Gonzalez MA, Ebrahim S, Bes-Rastrollo M, Nunez J, Martinez JA, et al Sedentary behaviors and the risk of incident hypertension: the SUN Cohort. American journal of hypertension. 2007;20(11):1156–62. Epub 2007/10/24. 10.1016/j.amjhyper.2007.06.007 . 17954361

[37] Mocanu V. Prevalence of overweight and obesity in urban elementary school children in northeastern Romania: its relationship with socioeconomic status and associated dietary and lifestyle factors. BioMed research international. 2013;2013:537451 Epub 2013/08/13. 10.1155/2013/537451 PubMed Central PMCID: PMCPmc3726018. 23936815 PMC3726018

[38] Thibault H, Carriere C, Langevin C, Kossi Deti E, Barberger-Gateau P, Maurice S. Prevalence and factors associated with overweight and obesity in French primary-school children. Public health nutrition. 2013;16(2):193–201. Epub 2012/09/08. 10.1017/s136898001200359x . 22953729 PMC10271746

[39] Takahashi H, Yoshika M, Yokoi T. Validation of two automatic devices: Omron HEM-7252G-HP and Omron HEM-7251G for self-measurement of blood pressure according to the European Society of Hypertension International Protocol revision 2010. Blood pressure monitoring. 2015 Epub 2015/05/02. 10.1097/mbp.0000000000000127 . 25932887

[40] Christofaro DGD, Fernandes RA, Gerage AM, Alves MJ, Polito MD, Oliveira ARd. Validação do monitor de medida de pressão arterial Omron HEM 742 em adolescentes. Arquivos brasileiros de cardiologia. 2009;92:10–5. 19219259 10.1590/s0066-782x2009000100003

[41] Chiolero A, Madeleine G, Gabriel A, Burnier M, Paccaud F, Bovet P. Prevalence of elevated blood pressure and association with overweight in children of a rapidly developing country. Journal of human hypertension. 2007;21(2):120–7. Epub 2006/12/01. 10.1038/sj.jhh.1002125 . 17136104

[42] Dong B, Wang Z, Song Y, Wang HJ, Ma J. Understanding trends in blood pressure and their associations with body mass index in Chinese children, from 1985 to 2010: a cross-sectional observational study. BMJ open. 2015;5(9):e009050 Epub 2015/09/13. 10.1136/bmjopen-2015-009050 ; PubMed Central PMCID: PMCPmc4567663. 26362667 PMC4567663

[43] Zhang YX, Wang M, Chu ZH, Xie L. Prevalence of relatively high blood pressure among children and adolescents with different body mass index and subcutaneous fat cut-offs. International journal of cardiology. 2015;179:536–8. Epub 2014/12/04. 10.1016/j.ijcard.2014.10.163 . 25466559

